# Spin on adverse effects in abstracts of systematic reviews of orthodontic interventions: a cross-sectional study (part 2)

**DOI:** 10.1186/s13643-023-02269-3

**Published:** 2023-06-20

**Authors:** Pauline A. J. Steegmans, Nicola Di Girolamo, Reint A. Meursinge Reynders

**Affiliations:** 1 Department of Orthodontics, Academisch Centrum Tandheelkunde Amsterdam (ACTA), University of Amsterdam, Gustav Mahlerlaan 3004, Amsterdam, 1081 LA The Netherlands; 2 Department of Clinical Sciences, College of Veterinary Medicine, Cornell University, 930 Campus Rd, Ithaca, NY 14853 USA; 3 Department of Oral and Maxillofacial Surgery, Academic Medical Center, University of Amsterdam, Meibergdreef 9, Amsterdam, 1105 AZ The Netherlands; 4 Studio Di Ortodonzia, Via Matteo Bandello 15, Milan, 20123 Italy

**Keywords:** Orthodontics, Reporting, Systematic review, Intervention, Spin, Misleading reporting, Misleading interpretation, Misleading extrapolation, Adverse effect, Adverse event, Harm, Safety

## Abstract

**Background:**

It is critical that abstracts of systematic reviews transparently report both the beneficial and adverse effects of interventions without misleading the readers. This cross-sectional study assessed whether adverse effects of interventions were reported or considered in abstracts of systematic reviews of orthodontic interventions and whether spin on adverse effects was identified when comparing the abstracts with what was sought and reported in these reviews.

**Methods:**

This cross-sectional study (part 2 of 2) used the same sample of 98 systematic reviews orthodontic interventions as used in part 1. Eligible reviews were retrieved from the Cochrane Database of Systematic Reviews and the 5 leading orthodontic journals between August 1 2009 and July 31 2021. Prevalence proportions were sought for 3 outcomes as defined in the published protocol. Univariable logistic regression models were built to explore associations between the presence of spin in the abstract and a series of predictors. Odds ratios (OR) 95% confidence intervals (95% CI) were used to quantify the strength of associations and their precision.

**Results:**

76.5% (75/98) of eligible reviews reported or considered (i.e., discussed, weighted etc.) potential adverse effects of orthodontic interventions in the abstract and the proportion of spin on adverse effects was 40.8% (40/98) in the abstract of these reviews. Misleading reporting was the predominant category of spin, i.e., 90% (36/40). Our explorative analyses found that compared to the Cochrane Database of Systematic Reviews all 5 orthodontic journals had similar odds of the presence of spin on adverse effects in abstracts of systematic reviews of orthodontic interventions. The odds of the presence of spin did not change over the sampled years (OR: 1.03, 95% CI: 0.9 to 1.16) and did not depend on the number of authors (OR: 0.93, 95% CI: 0.71 to 1.21), or on the type of orthodontic intervention (OR: 1.1, 95% CI: 0.45 to 2.67), or whether conflicts of interests were reported (OR: 0.74, 95% CI: 0.32 to 1.68).

**Conclusion:**

End users of systematic reviews of orthodontic interventions have to be careful when interpreting results on adverse effects in the abstracts of these reviews, because they could be jeopardized by uncertainties such as not being reported and misleading reporting as a result of spin.

**Supplementary Information:**

The online version contains supplementary material available at 10.1186/s13643-023-02269-3.

## Background

Abstracts should provide key information on a research study, which helps readers decide whether or not to access the full report [1]. It is therefore critical that abstracts transparently report the results of both the beneficial and adverse effects of interventions without misleading the readers. Misleading reporting, misleading interpretation, and misleading extrapolation of study results has been called “spin” [2, 3]. In this study, we assessed whether adverse effects of interventions were reported or considered in abstracts of both Cochrane and non-Cochrane reviews of orthodontic interventions and whether spin and what type of spin regarding adverse effects was present when comparing the abstracts with what was sought and reported in these reviews.

Titles and abstracts of publications of healthcare interventions are used for multiple purposes such as (1) an initial screening of the study type; (2) clarifying the included type of patients, interventions, comparators, outcomes, and settings; (3) obtaining a summary of the findings; and (4) an initial assessment of the validity of the study [1, 4, 5]. Titles and abstracts are the most and often only read sections of biomedical papers, because of a lack of time of readers, paywalls, or language issues [6]. It is therefore important that abstracts can be used as stand-alone documents that clearly and truthfully reflect what was reported in the full text [7]. The standard for Methodological Expectations of Cochrane Intervention Reviews (MECIR) [8] states under Item R13 that “The abstract of the review should aim to reflect a balanced summary of the benefits and harms of the intervention” and this is a “mandatory” Cochrane review standard. The inclusion of ‘adverse effects’ in this standard is crucial, because these effects are often poorly assessed and reported in clinical trials and systematic reviews of healthcare interventions [9–16].

In this context, it is important that findings on adverse effects are presented accurately in the abstract without misleading the reader. “A distorted presentation of study results” has been called “spin” [2, 3]. This definition and other commonly used definitions of spin and key terminology used in this article are listed in Table 1 [2, 3, 17–25]. Spin has been subdivided in 3 categories: “misleading reporting,” “misleading interpretations,” and “misleading extrapolations” of study results [2]. We adopted the definitions by Lazarus et al. [23] for these 3 categories of spin (Table 1). Controlling spin is important, because of its high prevalence and its consequences. For example, a randomized controlled trial (RCT) showed that spin in abstracts can influence the clinician’s interpretation of the results of a study [26]. Further, Yavchitz et al. [27] showed that the presence of spin in press releases and the mass media was related with spin in the conclusions of the pertinent abstracts of peer-reviewed RCTs. Our scoping searches showed that a high prevalence of spin has been recorded in abstracts of numerous research studies and for a wide variety of disciplines. For example, spin was present in 84% (107/128) of abstracts of reports of non-randomized studies assessing an intervention [23], 23% (24/105) of abstracts of RCTs in rheumatology [28], 57% (53/93) of abstracts of cardiovascular RCT reports [29], 34.2% (25/73) of abstracts of systematic reviews and meta-analyses related to treatment of proximal humerus fractures [30], 37.6% (27/72) of results, and 58.3% (42/72) of conclusions of abstracts of parallel-group RCTs with statistically non-significant results (*P* ≥ 0.05) [25]. Spin in abstracts of orthodontic studies was assessed in 2 recent publications [31, 32]. Guo et al. [31] found spin in 62.2% (69/111) of abstracts of parallel-group RCTs with clearly stated statistically non-significant primary outcomes and Makou et al. [32] identified spin in 48.6% (53/109) of abstracts of orthodontic meta-analyses.

**Table 1:** Glossary of terms

**Term**	**Definition**
Systematic review	Cochrane [17] defines a systematic review as follows: “A systematic review attempts to identify, appraise and synthesize all the empirical evidence that meets pre-specified eligibility criteria to answer a specific research question. Researchers conducting systematic reviews use explicit, systematic methods that are selected with a view aimed at minimizing bias, to produce more reliable findings to inform decision making.”
Intervention review	Cochrane [17] defines an intervention review as follows: “Intervention reviews assess the effectiveness/safety of a treatment, vaccine, device, preventative measure, procedure or policy.”
Orthodontic interventions	Steegmans et al. [18] define orthodontic interventions as follows: “Orthodontic interventions refer to the use of any type of orthodontic appliance to move teeth or change the jaw size or position for orthodontic purposes. These interventions also include appliances to maintain or stabilize the results of orthodontic treatment, for example retainers.”
Adverse effect	Cochrane [19, 20] defines an adverse effect as “an adverse event for which the causal relation between the intervention and the event is at least a reasonable possibility.”
Spin [3]	“Distorted presentation of study results.”
Spin [3]	“A misrepresentation of study results, regardless of motive (intentionally or unintentionally) that overemphasizes the beneficial effects of the intervention and overstates safety compared with that shown by the results.”
Spin [21]	“A specific intentional or unintentional reporting that fails to faithfully reflect the nature and range of findings and that could affect the impression the results produce in readers”
Spin [22]	“A specific reporting that fails to faithfully reflect the nature and range of findings and that could affect the impression that the results produce in readers, a way to distort science reporting without actually lying”
Misleading reporting related spin [23]	“Incomplete reporting of the study results that could be misleading for the reader.”
Misleading interpretation related spin [23]	Inadequate interpretation of the study results overestimating the beneficial effect of the intervention.
Misleading (inappropriate) extrapolation related spin [23]	Inappropriate generalization of the study results by inadequate (1) extrapolation from the population, interventions, or outcome actually assessed in the study to a larger population, different interventions, or outcomes or (2) inadequate implications for clinical practice.
Spin (in the abstract) on adverse effects of interventions [24]	Incomplete or inadequate reporting, interpretation, or extrapolation (or a combination of these variables) of findings on adverse effects of interventions in the abstract that could be misleading for the reader.

This is part 2 of 2 cross-sectional studies on assessing and reporting of adverse effects in systematic reviews of orthodontic interventions published in 5 leading orthodontic journals and in the Cochrane Database of Systematic Reviews. Part 1 focused predominantly on seeking and reporting of adverse effects in the main text and supplementary files of these reviews [18, 33]. In part 2, we assessed whether adverse effects of orthodontic interventions were reported or considered (i.e., discussed, weighed etc.) in abstracts of these reviews. We further measured whether spin was introduced in the abstract regarding information on adverse effects as found and reported in these reviews. We also assessed the different categories of spin. The findings of this research study are important not only for patients and clinicians but also for researchers, peer reviewers, and editors because they have a crucial role in reducing the prevalence of spin [34].

## Objectives

Our objectives were presented in the following 3 research questions [24]. Recent (up to October 31, 2021) scoping searches showed that these questions were not assessed previously.

- Question 1. In abstracts of systematic reviews of orthodontic interventions, were potential adverse effects of these interventions reported or considered (i.e., discussed, weighed etc.)?
- Question 2. Was spin identified on adverse effects of orthodontic interventions in the abstract?
- Question 3. What type of spin was identified on adverse effects of orthodontic interventions in the abstract?

## Methods

This manuscript reports the methods and results of part 2 of a cross-sectional study using the same 98 eligible reviews as in part 1 [33]. Additional information on the research methods and the characteristics of the included reviews can be found in part 1 [33] and in the published protocols of parts 1 and 2 [18, 24]. The protocol for this second cross-sectional study was published in “Research Integrity and Peer Review” [24] and can be consulted via the following link: https://researchintegrityjournal.biomedcentral.com/articles/10.1186/s41073-019-0084-4.

The checklist of the Strengthening the Reporting of Observational Studies in Epidemiology (STROBE) statement for cross-sectional studies [35] was included as Additional file 1. The differences between the methods planned in our protocol and those implemented in the final study were reported in Additional file 2. The rationales for these differences were also given. All raw data were reported in the Open Science framework Open Science Framework (https://osf.io/ka7mp/). There was no patient or public involvement during the development of the protocol or in the conduct of this study. The eligibility criteria, information sources, search strategy, and selection process used in part 1 of this cross-sectional study [33] were also used for part 2 of this study. To reduce the need of cross-checking between manuscripts, we reported these sections again.

### Eligibility criteria

The eligibility criteria were published previously in our protocol [24] and in part 1 of this study [33] and were developed by two researchers (PS and RMR). These criteria are presented in Table 2 [36] and are further explained under here.

**Table 2:** Eligibility criteria

**Item**	**Included**	**Excluded**
**Study designs**	Systematic reviews of orthodontic interventions. The definition of systematic review, intervention review, and orthodontic interventions listed in the Glossary of terms will be used to assess whether a review is eligible (Table 1).	1) Noninterventional reviews such as “Methodology,” “Diagnostic,” “Qualitative,” and “Prognostic” 2) Rapid and scoping reviews 3) Systematic reviews with Bayesian network meta-analysis 4) Systematic reviews of interventions that did not find any eligible studies (empty reviews)
**Participants**	Systematic reviews on any type of patients undergoing orthodontic interventions, i.e., patients of any health status, sex, age, and demographics, and socio-economic status.	1) Intervention reviews that focus exclusively on patients with congenital anomalies, for example with cleft lip and palate 2) Systematic reviews of animal or laboratory studies
**Interventions**	1) Systematic reviews that assessed the effects of clinical orthodontic interventions. Clinical orthodontic interventions refer to any type of orthodontic appliance that are used to move teeth or change the jaw size or position for orthodontic purposes 2) Systematic reviews of interventions with appliances to maintain or stabilize the outcomes of orthodontic treatment, for example retainers 3) Systematic reviews of orthodontic interventions that compared the effects of orthodontic treatment with or without additional interventions such as pharmacological or small surgical interventions, e.g., periodontal or implant surgery 4) No exclusion criteria were applied to the characteristics of the operator who conducted the interventions	1) Systematic reviews in which patients receive orthodontic treatment, but in which the effects of other interventions, e.g., periodontal surgery, were compared and not the effects of orthodontic interventions 2) Systematic reviews of interventions in which orthodontic appliances were specifically used for other purposes, e.g., changing jaw positions to treat respiration or temporomandibular disorders 3) Systematic review of orthodontic interventions that included orthognathic surgery 4) Systematic reviews that focused exclusively on adverse effects of interventions 5) Systematic reviews that did not assess a specific orthodontic intervention but referred to orthodontic treatment as a whole
**Outcomes**	1) Any adverse effect of orthodontic interventions scored at any endpoint or timing 2) The effects of orthodontic interventions did not refer just to outcomes related to tooth and jaw size and positions but also to broader outcomes such as periodontal health, esthetic changes, the health of the temporomandibular joint, patient health experiences, and economic issues associated with the interventions 3) The reporting of outcomes on adverse effects did not determine eligibility of reviews for this cross-sectional study, i.e., reviews were not excluded because they did not report measured outcome data in a “usable” way [36].	No exclusion criteria
**Stetting**	Any type of setting in which the interventions were conducted, i.e., university or private practice etc.	No exclusion criteria

#### Study designs

- We included systematic reviews of orthodontic interventions. The definitions of the terms “systematic review,” “intervention review,” and “orthodontic interventions” listed in the Glossary of terms (Table 1) were used to assess eligibility.
- The following reviews were excluded: (1) noninterventional reviews such as “Methodology,” “Diagnostic,” “Qualitative,” and “Prognostic”; (2) rapid and scoping reviews; (3) systematic reviews with Bayesian network meta-analysis; and (4) systematic reviews of interventions that did not find any eligible studies (empty reviews).

#### Participants

- Systematic reviews of interventions on any type of patients undergoing orthodontic interventions, i.e., patients of any health status, sex, age, and demographics, and socio-economic status were eligible.
- Intervention reviews that focused exclusively on patients with congenital anomalies, for example, with cleft lip and palate and systematic reviews of animal or laboratory studies were excluded.

#### Interventions

- Systematic reviews on the following interventions were eligible: (1) systematic reviews that assessed the effects of clinical orthodontic interventions. Clinical orthodontic interventions refer to any type of orthodontic appliance that are used to move teeth or change the jaw size or position for orthodontic purposes; (2) systematic reviews of interventions with appliances to maintain or stabilize the outcomes of orthodontic treatment, for example, retainers; (3) systematic reviews of orthodontic interventions that compared the effects of orthodontic treatment with or without additional interventions such as pharmacological or small surgical interventions, e.g., periodontal or implant surgery; and (4) no exclusion criteria were applied to the characteristics of the operator who conducted the interventions.
- Systematic reviews on the following interventions were excluded: (1) systematic reviews in which patients receive orthodontic treatment, but in which the effects of other interventions, e.g., periodontal surgery, were compared and not the effects of orthodontic interventions; (2) systematic reviews of interventions in which orthodontic appliances were specifically used for other purposes, e.g., changing jaw positions to treat respiration or temporomandibular disorders; (3) systematic review of orthodontic interventions that included orthognathic surgery; (4) systematic reviews that focused exclusively on adverse effects of interventions; and (5) systematic reviews that did not assess a specific orthodontic intervention but referred to orthodontic treatment as a whole.

#### Outcomes

- Systematic reviews of orthodontic interventions that assessed any adverse effect of orthodontic interventions scored at any endpoint or timing were eligible. The effects of orthodontic interventions did not refer just to outcomes related to tooth and jaw size and positions but also to broader outcomes such as periodontal health, esthetic changes, the health of the temporomandibular joint, patient health experiences, and economic issues associated with the interventions. The reporting of outcomes on adverse effects did not determine eligibility of reviews for this cross-sectional study, i.e., reviews were not excluded because they did not report measured outcome data in a “usable” way [36].
- No exclusion criteria regarding the outcomes of systematic reviews of orthodontic interventions were applied.

#### Setting

- Systematic reviews of orthodontic interventions that reported on interventions conducted in any type of setting, i.e., university or private practice, were eligible.

### Information sources and search strategy

The Cochrane Database of Systematic reviews [37] and the websites of 5 leading orthodontic journals were the information sources of this study. The journal selection of the latter journals was based on two criteria: (1) the journal has been published for 10 years or more and (2) the highest impact factor. The following 5 orthodontic journals fulfilled these criteria: European Journal of Orthodontics [EJO], American Journal of Orthodontics and Dentofacial Orthopedics [AJODO], Angle Orthodontist (AO), The Korean Journal of Orthodontics (KJO), and Orthodontics and Craniofacial Research (OCR). These journals were manually searched from August 1, 2009, until July 31, 2021, for systematic reviews that fulfilled the eligibility criteria. August 1, 2019, was chosen as the starting date, because it coincides with the launch of the Preferred Reporting Items for Systematic reviews and Meta-Analyses (PRISMA) statement on 21 July 2009 [38, 39].

### Study records

#### Selection process

Two reviewers (PS and RMR) manually searched systematic reviews that fulfilled the eligible criteria. Pilot tests were conducted a priori to train both reviewers and to calibrate them. All titles and abstracts in the websites of the 5 orthodontic journals were hand-searched for eligible reviews. Eligible Cochrane reviews were searched in the section “Dentistry and Oral health” in the Cochrane library. Only the latest version of a review was eligible when review updates had been published. In the case of disagreement on the selection procedures, the following strategies were implemented and in this sequence: (1) discussions between these operators, (2) rereading the paper, (3) contacting of authors by email to clarify issues regarding a specific manuscript. Persistent disagreements were resolved through consultation with a methodologist. A total of 98 eligible systematic reviews of orthodontic interventions was identified in part 1 of this study [33]. This same sample of 98 studies was also used in this study.

#### Data collection procedures

All 98 eligible reviews together with their supplemental files were merged into binder PDFs, and according to protocol [24], pertinent search terms were linked to these documents to facilitate data extraction (Additional file 2). Our pilot-tested data collection forms were used to extract data and are given in Additional file 2. Data items were collected from the entire eligible review, i.e., the entire manuscript including the abstract, tables, figures, and additional files. We implemented this procedure for all eligible reviews but did not extract data from the plain language summary of eligible Cochrane reviews. Two calibrated authors (PS and RMR) independently collected data from the 98 eligible reviews to address the research questions. In the case of disagreement, we applied the same strategies as reported in the section “Selection process” and the third author (NDG) was consulted in the case of persistent disagreements.

#### Assessing adverse effects of orthodontic interventions

Pain and the various categories of adverse effects hypothetically linked to orthodontic interventions as defined by Preoteasa et al. [40] and modified by Steegmans et al. [33] were reported in a table in Additional file 2. This table was consulted as our reference to assess the reporting on adverse effects in the abstract. When additional adverse effects were identified that were not given in this table, we included them with rationale. Effects that could be labeled either as “beneficial” or “adverse” were not included unless the review authors labeled these ambiguous effects as “adverse.” Explanations for such decisions were given. Orthodontic interventions were classified in three types, i.e., type 1: orthodontic interventions to move teeth or change the jaw size or position for orthodontic purposes, type 2: orthodontic interventions with additional surgical, pharmacological, or vibratory interventions, and type 3: orthodontic interventions to maintain or stabilize orthodontic results.

#### Assigning spin of adverse effects of orthodontic interventions in abstracts of systematic reviews

Spin was assigned by comparing whether what was reported in the abstract on adverse effects of orthodontic interventions was congruent with the findings on these effects in the review. Three types of spin were assigned i.e., misleading reporting, misleading interpretation, and misleading (inappropriate) extrapolation on adverse effects of orthodontic interventions in abstracts of systematic reviews [27]. To facilitate this assignment and to reduce the risk of misinterpretation, each type of spin was subdivided in categories. The presence of spin was assigned when it was identified in one or more of these categories. Spin was assessed in all eligible reviews irrespective of whether these reviews sought adverse effects of interventions or not. Because the pilot tests for our protocol identified only 2 reviews with spin [24] and because assessing spin is not easy [22], we conducted additional pilot tests on 10 RCTs to further calibrate the operators (PS and RMR) that assigned spin and to fine-tune the descriptions of spin and the checklists for assigning spin. These fine-tuned descriptions of the different types of spin and the pertinent data collection forms to identify spin are reported respectively in Table 3 and Additional file 2. Definitions of spin were given for reviews that sought and those that did not seek adverse effects of orthodontic interventions (Table 3).

**Table 3:** Types of spin in reviews that did or did not seek adverse effects of interventions

**Definitions of the 3 types of spin**	**Reviews that sought adverse effects of interventions**	**Reviews that did not seek adverse effects of interventions**
**Misleading reporting (in the abstract) on adverse effects of interventions:** “Incomplete or inadequate reporting in the abstract on the results of adverse effects that were not supported by the findings of the review.”	**Categories:** 1) Not reporting in the abstract on the results of adverse effects found in the review. 2) Selective reporting in the abstract on the results of adverse effects found in the review.	**Categories:** 1) Reporting on results of adverse effects in the abstract when adverse effects were not sought. 2) Reporting in the abstract that adverse effects were sought when they were not sought.
**Misleading interpretation (in the abstract) on adverse effects of interventions:** “Interpretation in the abstract on the results of adverse effects that was not supported by the findings of the review.” This could for example underestimated the adverse effects of the intervention.	**Categories:** 1) Claiming in the abstract that the intervention is safe (has no or minimal adverse effects), despite concerning results on adverse effects found in the review, e.g., based on non-statistically significant results on adverse effects with wide confidence intervals [27]. 2) Downgrading in the abstract the importance of the adverse effects, despite concerning results on adverse effects found in the review. 3) Recommendations are made in the abstract for clinical practice that are not supported by the findings in the review on adverse effects [27].	**Categories:** 1) Claiming in the abstract that the intervention is safe (has no or minimal adverse effects) despite not having sought adverse effects. 2) Downgrading in the abstract the importance of the adverse effects, despite not having sought adverse effects. 3) Recommendations are made in the abstract for clinical practice despite not having sought adverse effects.
**Misleading (inappropriate) extrapolation (in the abstract) on adverse effects of interventions:** “Overgeneralisation in the abstract of the study results to different populations, interventions, outcomes or settings than were assessed in the review despite evidence on adverse effects on a different population, intervention, outcome or setting.”	**Categories:** 1) Results are extrapolated in the abstract to another population, intervention, outcome, or setting than were assessed in the review despite evidence on adverse effects on a different population, intervention, outcome, or setting.	**Categories:** 1) Results are extrapolated in the abstract to another population, intervention, outcome, or setting than were assessed in the review despite not having sought adverse effects.

#### Power calculation

In our pilot sample, we identified an overall proportion of 14.3% (2/14) of spin of the adverse effects in the abstracts of 14 systematic reviews of orthodontic interventions. For this proportion, the Epitools software [41] calculated a required sample size of 48 studies (precision 0.1 and confidence level 0.95), which was fulfilled by our 98 eligible reviews.

### Outcomes and statistical analyses

#### Outcomes

Prevalence proportions were calculated to quantify the answers to our 3 research questions in the 98 selected reviews. According to our published protocol, these proportions were also calculated separately for reviews that either did (*n* = 84) or did not (*n* = 14) seek any findings related to adverse effects of interventions in the included studies [33]. These statistics were calculated for (1) all journals as a one group, (2) the five leading orthodontic journals together and the Cochrane Database of Systematic Reviews separately, and (3) each eligible journal separately.

#### Explorative analyses

Univariable logistic regression models were built to determine the association between the presence of spin in the abstract and characteristics of the systematic review, i.e., journal, year of publication, number of authors, conflict of interest reported, conflict of interest present, funding reported, and type of orthodontic intervention. These analyses were not registered in the protocol and should therefore be interpreted as exploratory. The strengths of associations were quantified using odds ratios (OR) and 95% confidence intervals (95% CI). Multivariable models were built if multiple significant predictors were found in the univariable analysis. Analyses were performed with the use of commercial software (IBM SPSS 22.0, SPSS Inc, Chicago, IL). A two-sided *P* value of 0.05 was considered to be statistically significant.

## Results

**Fig. 1:**
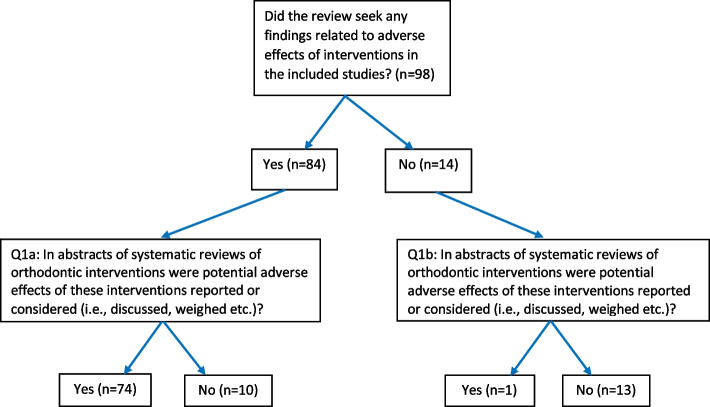
Reporting or considering adverse effects of orthodontic interventions in abstracts of systematic reviews

**Fig. 2:**
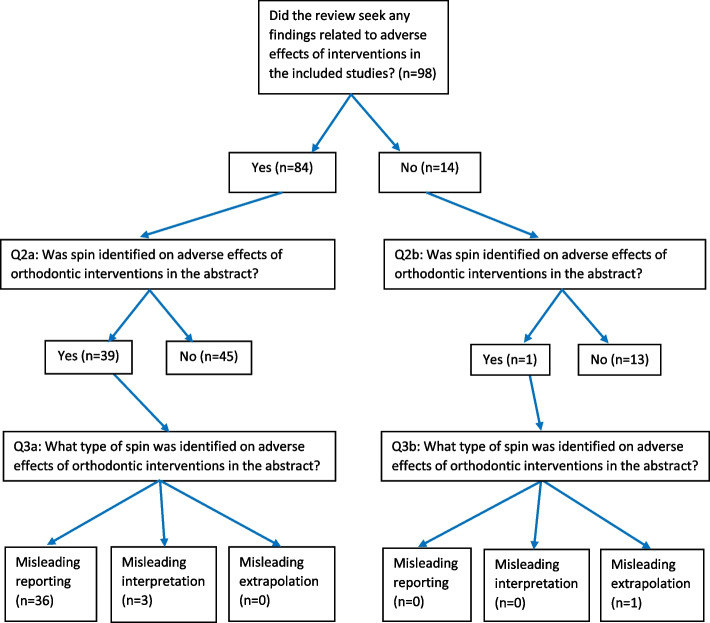
Spin on adverse effects of orthodontic interventions in abstracts of systematic reviews

The results of the search for eligible reviews were reported previously in part 1 of this cross-sectional study [33] and identified 98 eligible reviews. The PRISMA flow diagram and all included reviews and all excluded studies with rationale were given again in Additional file 3. Figures 1 and 2 present the flow diagrams of the answers to the research questions, and Tables 4 and 5 report the pertinent proportion statistics. The number of identified systematic reviews and the number of eligible systematic reviews of orthodontic interventions given in these tables were published previously [33] and were reported again to give context to the outcomes to our research questions. In these tables, outcomes are further subdivided for eligible reviews that did (*n* = 84) or did not (*n* = 14) seek any findings related to adverse effects of interventions in the included studies. The initial inter-operator agreement between both operators for assigning spin was high (Cohen’s *κ* = 0.94), and complete agreement between operators was reached after discussion.

**Table 4:** Outcomes on reporting or considering adverse effects and spin in abstracts of systematic reviews of orthodontic interventions

**Description of outcomes**	**Cochrane**	**EJO**	**AJODO**	**AO**	**KJO**	**O&CR**	**All orthodontic journals**	**All journals**
The number of identified systematic reviews	*n* = 29	*n* = 100	*n* = 68	*n* = 61	*n* = 11	*n* = 53	*n* = 293	*n* = 322
The number of eligible systematic reviews of orthodontic interventions	*n* = 10	*n* = 28	*n* = 20	*n* = 23	*n* = 3	*n* = 14	*n* = 88	*n* = 98
The prevalence of eligible systematic reviews of orthodontic interventions that sought any findings related to adverse effects of interventions in the included studies^a^	100.0% (10/10)	92.9% (26/28)	75.0% (15/20)	78.3% (18/23)	100.0% (3/3)	85.7% (12/14)	84.1% (74/88)	85.7% (84/98)
Outcome 1: The prevalence of eligible systematic reviews in which potential adverse effects of interventions were reported or considered (i.e., discussed, weighted etc.) in the abstract^a^	100.0% (10/10)	82.1% (23/28)	65.0% (13/20)	65.2% (15/23)	100.0% (3/3)	78.6% (11/14)	73.9% (65/88)	76.5% (75/98)
Outcome 1a: The prevalence of eligible systematic reviews in which potential adverse effects of interventions were reported or considered (i.e., discussed, weighted etc.) in the abstract^a^	100% (10/10)	88.5% (23/26)	86.7% (13/15)	77.8% (14/18)	100% (3/3)	91.7% (11/12)	86.5% (64/74)	88.1% (74/84)
Outcome 1b: The prevalence of eligible systematic reviews in which potential adverse effects of interventions were reported or considered (i.e., discussed, weighted etc.) in the abstract^a^	00.0% (0/0)	0.0% (0/2)	0.0% (0/5)	20.0% (1/5)	0.0% (0/0)	0.0% (0/2)	7.1% (1/14)	7.1% (1/14)
Outcome 2: The prevalence of eligible systematic reviews in which spin was identified on adverse effects of orthodontic interventions in the abstract^a^	60.0% (6/10)	35.7% (10/28)	30.0% (6/20)	39.1% (9/23)	66.7% (2/3)	50.0% (7/14)	38.6% (34/88)	40.8% 40/98
Outcome 2a: The prevalence of eligible systematic reviews in which spin was identified on adverse effects of orthodontic interventions in the abstract^a^	60.0% (6/10)	38.5% (10/26)	40.0% (6/15)	44.4% (8/18)	66.7% (2/3)	58.3% (7/12)	44.6% (33/74)	46.4% (39/84)
Outcome 2b: The prevalence of eligible systematic reviews in which spin was identified on adverse effects of orthodontic interventions in the abstract^a^	0.0% (0/0)	0.0% (0/2)	0.0% (0/5)	20.0% (1/5)	0.0% (0/0)	0.0% (0/2)	7.1% (1/14)	7.1% (1/14)

^a^Outcome 1a addresses research question 1a, outcome 1b addresses research question 1b, and outcome 1 addresses the answers to questions 1a and 2b combined, outcome 2a addresses research question 2a, outcome 2b addresses research question 2b, and outcome 2 addresses the answers to questions 2a and 2b combined (see Figs. 1 and 2)

**Table 5:** Outcome 3: types and categories of spin in abstracts of systematic reviews of orthodontic interventions

**Type of spin and category**	**Cochrane**	**EJO**	**AJODO**	**AO**	**KJO**	**O&CR**	**All orthodontic journals**	**All journals**
**Misleading reporting** **Category:** Not reporting in the abstract on the results of adverse effects found in the review.	*n* = 2	*n* = 3	*n* = 2	*n* = 5	*n* = 0	*n* = 1	*n* = 11 Prevalence: 32.4% (11/34)	*n* = 13 Prevalence: 32.5% (13/40)
**Misleading reporting** **Category:** Selective reporting in the abstract on the results of adverse effects found in the review	*n* = 4	*n* = 6	*n* = 4	*n* = 2	*n* = 2	*n* = 5	*n* = 19 Prevalence: 55.9% (19/34)	*n* = 23 Prevalence: 57.5% (23/40)
**Misleading interpretation** **Category:** Claiming in the abstract that the intervention is safe (has no or minimal adverse effects), despite concerning results on adverse effects found in the review		*n* = 1		*n* = 1		*n* = 1	*n* = 3 Prevalence: 8.8% (3/34)	*n* = 3 Prevalence: 7.5% (3/40)
**Misleading (inappropriate) extrapolation** **Category:** Results are extrapolated in the abstract to another population, intervention, outcome or setting than were assessed in the review despite evidence on adverse effects on a different population, intervention, outcome or setting.				*n* = 1			*n* = 1 Prevalence: 2.9% (1/34)	*n* = 1 Prevalence: 2.5% (1/40)

### Results for questions 1a and 1b

The results for questions 1a and 1b combined showed that the majority 76.5% (75/98) of eligible reviews reported or considered (i.e., discussed, weighted etc.) potential adverse effects of orthodontic interventions in the abstract (Fig. 1). This prevalence was much higher in the reviews that sought any findings related to adverse effects of interventions in the included studies (88.1% (74/84) than those reviews that did not seek these findings (7.1% (1/14) (Fig. 1).

### Results for questions 2a and 2b

The results for questions 2a and 2b combined showed that the total proportion of the presence of spin on adverse effects in the abstract was 40.8% (40/98) in the eligible reviews (Table 4). This prevalence was considerable higher in the reviews that sought any findings related to adverse effects of interventions in the included studies (Question 2a), i.e., 46.4% (39/84) than those reviews that did not seek such findings 7.1% (1/14) (Fig. 2).

### Results for questions 3a and 3b

For questions 3a and 3b combined, misleading reporting was the predominant type of spin i.e., 90% (36/40), which was subdivided in the categories of not reporting, 32.5% (13/40), and selective reporting 57.5% (23/40) (Table 5). Misleading interpretation and misleading (inappropriate) extrapolation types of spin were respectively 7.5% (3/40) and 2.5% (1/40).

### Explorative analyses

The findings of our explorative analysis on the presence of spin on adverse effects in abstracts of systematic reviews of orthodontic interventions were reported in Table 6. Compared to the Cochrane Database of Systematic Reviews, the EJO (OR: 0.37, 95% CI: 0.08 to 1.63), the AJODO (OR: 0.29, 95% CI: 0.06 to 1.39), the AO (OR: 0.43, 95% CI: 0.09 to 1.95), the KJO (OR: 1.33, 95% CI: 0.09 to 20.11), and the O&C (OR: 0.67, 95% CI: 0.13 to 3.45) had similar odds of the presence of spin on adverse effects in abstracts of systematic reviews of orthodontic interventions. The odds of the presence of spin on adverse effects in abstracts of systematic reviews of orthodontic interventions did not change over the sampled years (OR: 1.03, 95% CI: 0.9 to 1.16). The odds of the presence of spin on adverse effects in abstracts of systematic reviews of orthodontic interventions did not change depending on the number of authors (OR: 0.93, 95% CI: 0.71 to 1.21). Compared to systematic reviews that did not report conflicts of interest, systematic reviews that reported conflicts of interest had similar odds (OR: 0.74, 95% CI: 0.32 to 1.68) of the presence of spin on adverse effects in abstracts of systematic reviews of orthodontic interventions. Systematic reviews on type 1 orthodontic interventions, i.e., orthodontic interventions to move teeth or change the jaw size or position for orthodontic purposes compared with systematic reviews on type 3 orthodontic interventions, i.e., orthodontic interventions to maintain or stabilize orthodontic results had similar odds (OR: 1.1, 95% CI: 0.45 to 2.67) of the presence of spin on adverse effects of orthodontic interventions in abstracts of systematic reviews (Table 6). Intervention type 2, i.e., orthodontic interventions with additional surgical, pharmacological, or vibratory interventions was not included in the analysis since there was only 1 systematic review of this type of intervention.

**Table 6:** Associations between presence of spin in the abstract and characteristics of the systematic review

**Item**	**Variable insertion in the model**	**Description**	**No (%)**	**Yes (%)**	**OR**	**Lower 95%CI**	**Upper 95%CI**	***P* value**
**Journal**	**Categorical**	Cochrane	4 (40.0%)	6 (60.0%)	1	-	-	-
		EJO	18 (64.3%)	10 (35.7%)	0.37	0.08	1.63	0.19
		AJODO	14 (70.0%)	6 (30.0%)	0.29	0.06	1.39	0.12
		AO	14 (60.9%)	9 (39.1%)	0.43	0.09	1.95	0.27
		KJO	1 (33.3%)	2 (66.7%)	1.33	0.09	20.11	0.84
		O&C	7 (50.0%)	7 (50.0%)	0.67	0.13	3.45	0.63
**Year of publication**	**Continuous**				1.03	0.9	1.16	0.7
		2009	1 (100.0%)	0 (0.0%)				
		2010	1 (33.3%)	2 (66.7%)				
		2011	5 (71.4%)	2 (28.6%)				
		2012	2 (100.0%)	0 (0.0%)				
		2013	6 (66.7%)	3 (33.3%)				
		2014	3 (33.3%)	6 (66.7%)				
		2015	9 (75.0%)	3 (25.0%)				
		2016	5 (55.6%)	4 (44.4%)				
		2017	5 (55.6%)	4 (44.4%)				
		2018	6 (46.2%)	7 (53.8%)				
		2019	3 (75.0%)	1 (25.0%)				
		2020	7 (58.3%)	5 (41.7%)				
		2021	5 (62.5%)	3 (37.5%)				
**Number of authors**	**Continuous**				0.93	0.71	1.21	0.59
		2	3 (50.0%)	3 (50.0%)				
		3	7 (50.0%)	7 (50.0%)				
		4	16 (69.6%)	7 (30.4%)				
		5	15 (55.6%)	12 (44.4%)				
		6	8 (53.3%)	7 (46.7%)				
		7	6 (66.7%)	3 (33.3%)				
		8	2 (100.0%)	0 (0.0%)				
		9	1 (50.0%)	1 (50.0%)				
**Conflict of interest reported**	**Categorical**	Yes	32 (56.1%)	25 (43.9%)	0.74	0.32	1.68	0.47
		No	26 (63.4%)	15 (36.6%)	1	-	-	-
**Conflict of interest present**		Not reported	26 (63.4%)	15 (36.6%)	NA	NA	NA	NA
		No	32 (56.1%)	25 (43.9%)	NA	NA	NA	NA
**Funding reported**	**Categorical**	Yes	23 (56.1%)	18 (43.9%)	0.8	0.35	1.81	0.6
		No	35 (61.4%)	22 (38.6%)	1	-	-	-
**Type of orthodontic intervention**^**a**^	**Categorical**	1	41 (59.4%)	28 (40.6%)	1	-	-	-
		2	1 (100.0%)	0 (0.0%)	NA	NA	NA	NA
		3	16 (57.1%)	12 (42.9%)	1.1	0.45	2.67	0.84

^a^Type 1 orthodontic interventions: Orthodontic interventions to move teeth or change the jaw size or position for orthodontic purposes. Type 2 orthodontic interventions: Orthodontic interventions with additional surgical, pharmacological, or vibratory interventions. Type 3 orthodontic interventions: Orthodontic interventions to maintain or stabilize orthodontic results

## Discussion

### Principal findings of the study

This cross-sectional study showed that the majority, i.e., 76.5% (75/98), of the eligible systematic reviews reported or considered (i.e., discussed, weighted) potential adverse effects of orthodontic interventions in the abstract (Table 4). In 40.8% (40/98), spin on adverse effects was found in the abstract of these reviews (Table 4). Spin related to misleading reporting was the predominant, i.e., 90.0% (36/40), type of spin (Table 5). No association was found between the presence of spin in the abstract and any of the predictors (Table 6).

### Comparison with other studies

Item 1 of the 2004 CONSORT (Consolidated Standards of Reporting Trials) Harms extension [42] states that “if the study collected data on harms and benefits, the title or abstract should so state”. In this second part of our cross-sectional studies, 76.5% (75/98) of systematic reviews reported or considered adverse effects of interventions in the abstracts. This was lower than the 84.7% (83/98) of reviews that sought and reported findings on adverse effects in the main text or supplementary files of these reviews as we reported in part 1 [33]. A recent overview of systematic reviews by Junqueira et al. [43] found that harms were reported in 47% (258/552) of the abstracts of RCTs published prior to the CONSORT harms statement and in 54% (643/1201) of the abstracts of the RCTs published after the publication of this statement, indicating only a limited improvement in recent years. Qureshi et al. [14] found that most systematic reviews 81.4% (57/70) on interventions with gabapentin reported a statement on harms in the abstract. Different results in reporting of adverse effects in abstracts in this cross-sectional study compared with those in other studies could be the result of variables such as differences in (1) research design, i.e., systematic reviews versus RCTs, (2) sample size, (3) what is reported on harms in the abstract, e.g., specific versus more general statements, and (4) the field of research. For example, the relatively high prevalence of reporting of adverse effects in abstracts of systematic reviews in this study could be the result of having included only reviews of orthodontic interventions. Orthodontists might be more attentive in assessing adverse effects, because assessing adverse events such as undesired outcomes of orthodontic treatment and relapse is part of daily clinical practice.

A wide variety of prevalence proportions on spin has been identified in abstracts of systematic reviews of randomized-and non-randomized studies [23, 25, 28–30]. These studies identified proportions of spin that varied between 23% (24/105) of spin in abstracts of RCTs in rheumatology [28] and 84% (107/128) of spin in abstracts of non-randomized studies that assessed interventions [23]. According to our scoping searches, spin in the field of orthodontics has been assessed only in 2 recent studies [31, 32]. Spin was identified in 62.2% (69/111) of abstracts of parallel-group RCTs with clearly stated statistically non-significant primary outcomes [31] and in 48.6% (53/109) of abstracts of orthodontic meta-analyses [32]. In our study, none of the predictors assessed was associated with the presence of spin in this study. Similar findings were identified by Guo et al. [31] on the overlapping predictors with our study, i.e., “the year of publication” and “the number of authors.” Makou et al. [32] also found no association with the presence of spin for the overlapping predictors “journal” and “year of publication” but found a higher risk of spin in studies with a large number of authors (≥ 6). However, direct comparisons of our results on spin and those identified in other studies are often difficult because of differences in variables such as (1) the types and subtypes of spin and definitions of spin, (2) the research design, (3) the locations in the text where spin was assessed, (4) the field of research, (5) the types of interventions, (6) the journals included, and (7) the time point of the publication [42].

### Strengths and limitations

This study has the following strengths: (1) the research methods were pilot tested on a series of systematic reviews and RCTs to consistently extract data and to calibrate data extractors; (2) the protocol of this study was published a priori; and (3) according to our scoping searches, this is the first study that assessed spin on adverse effects in abstracts of systematic reviews of interventions. These searches also showed that our protocol was the first article [24] that planned to assess spin in the field of orthodontics. Subsequently, 2 additional studies [31, 32] have assessed other types of spin in the orthodontic literature, indicating a growing interest in this topic. (4) All raw data were either included with this manuscript in additional files or registered in Open Science Framework (https://osf.io/ka7mp/).

This study also has limitations such as (1) the risk of inaccurate findings on reporting adverse effects of interventions in abstracts as a result of the inconsistent assessment and reporting of adverse effects in both primary research and in systematic reviews and (2) the assessment of spin is not completely objective [44]. However, our inter-operator agreement was high as indicated by a high Cohen’s *κ* (0.94), and disagreements were completely resolved through discussions. (3) The wide variety of different types and definitions of spin and the assessment of spin in different contexts often limits comparing findings on spin between studies [44]. (4) This study assessed a variety of proportions exclusively in Cochrane intervention reviews and in the 5 orthodontic journals with the highest impact factor. Our findings therefore probably underestimate the true magnitude of proportions on poor reporting and spin on adverse effects in the abstracts of the wider body of orthodontic systematic reviews, and (5) the true magnitude of some proportions could also be underestimated, because we assessed a recent sample (August 1, 2009, until July 31, 2021) of reviews. In this context, one should consider that poor reporting has decreased over time as was shown in a study that assessed the evolution of poor reporting in 20,920 RCTs included in a sample of Cochrane reviews [45].

### Implications and future research

Our results imply that end-users of systematic reviews of orthodontic interventions have to be careful when interpreting the findings on adverse effects in abstracts of both Cochrane reviews and those published in the 5 leading orthodontic journals. This is particularly important, because the title and abstracts are often the only read sections of biomedical papers [6] and spin in abstracts can bias the clinician’s interpretations of the results [26]. Reading the full text of research studies is not a solution, because recent studies showed that the proportions of spin in abstracts are similar to those in the full text of the pertinent RCTs [29] and systematic reviews [46]. Guideline developers, researchers, peer reviewers, and editors have an important role in tackling poor reporting and spin regarding adverse effects in abstracts of systematic reviews of orthodontic interventions. Standards for reporting adverse effects in abstracts of systematic reviews of interventions have to be developed. Much research is ahead.

### Supplementary Information

**Additional file 1.** STROBE Checklist: Checklist of items that should be included in reports of cross-sectional studies [35].

**Additional file 2.** 2A. Differences between the protocol and the completed cross-sectional study. 2B. Search terms and their derivatives. 2C. Data collection forms. 2D. Adverse effects hypothetically linked to orthodontic interventions.

**Additional file 3.** 3A. PRISMA flow diagram. 3B. Included reviews. 3C. Excluded studies with rationale

## Data Availability

Not applicable.

## Abbreviations

AJODO: American Journal of Orthodontics and Dentofacial Orthopedics
AO: Angle Orthodontist
EJO: European Journal of Orthodontics
KJO: Korean Journal of Orthodontics
O&CR: Orthodontics and Craniofacial Research
MECIR: Methodological Expectations of Cochrane Intervention Reviews
PRISMA: Preferred reporting items for systematic reviews and meta-analyses
RCT: Randomized controlled trial
STROBE: Strengthening the Reporting of Observational Studies in Epidemiology
