# Correction: Spin on adverse effects in abstracts of systematic reviews of orthodontic interventions: a cross-sectional study (part 2)

**DOI:** 10.1186/s13643-024-02509-0

**Published:** 2024-03-19

**Authors:** Pauline A. J. Steegmans, Nicola Di Girolamo, Reint A. Meursinge Reynders

**Affiliations:** 1 Department of Orthodontics, Academisch Centrum Tandheelkunde Amsterdam (ACTA), University of Amsterdam, Gustav Mahlerlaan 3004, Amsterdam, 1081 LA The Netherlands; 2 Department of Clinical Sciences, College of Veterinary Medicine, Cornell University, 930 Campus Rd, Ithaca, NY 14853 USA; 3 Department of Oral and Maxillofacial Surgery, Academic Medical Center, University of Amsterdam, Meibergdreef 9, Amsterdam, 1105 AZ The Netherlands; 4 Studio Di Ortodonzia, Via Matteo Bandello 15, Milan, 20123 Italy

**Correction** **:** **Syst Rev 12, 99 (2023)**

10.1186/s13643-023-02269-3

Following publication of the original article [1], the authors reported a typo error in Table 6 wherein the headings ‘Yes’ and ‘No’ were interchanged. The correct table is given below.

Incorrect Table 6:

**Table 6:** Associations between presence of spin in the abstract and characteristics of the systematic review

**Item**	**Variable insertion in the model**	**Description**	**Yes (%)**	**No (%)**	**OR**	**Lower 95%CI**	**Upper 95%CI**	***P* value**
**Journal**	**Categorical**	Cochrane	4 (40.0%)	6 (60.0%)	1	-	-	-
		EJO	18 (64.3%)	10 (35.7%)	0.37	0.08	1.63	0.19
		AJODO	14 (70.0%)	6 (30.0%)	0.29	0.06	1.39	0.12
		AO	14 (60.9%)	9 (39.1%)	0.43	0.09	1.95	0.27
		KJO	1 (33.3%)	2 (66.7%)	1.33	0.09	20.11	0.84
		O&C	7 (50.0%)	7 (50.0%)	0.67	0.13	3.45	0.63
**Year of publication**	**Continuous**				1.03	0.9	1.16	0.7
		2009	1 (100.0%)	0 (0.0%)				
		2010	1 (33.3%)	2 (66.7%)				
		2011	5 (71.4%)	2 (28.6%)				
		2012	2 (100.0%)	0 (0.0%)				
		2013	6 (66.7%)	3 (33.3%)				
		2014	3 (33.3%)	6 (66.7%)				
		2015	9 (75.0%)	3 (25.0%)				
		2016	5 (55.6%)	4 (44.4%)				
		2017	5 (55.6%)	4 (44.4%)				
		2018	6 (46.2%)	7 (53.8%)				
		2019	3 (75.0%)	1 (25.0%)				
		2020	7 (58.3%)	5 (41.7%)				
		2021	5 (62.5%)	3 (37.5%)				
**Number of authors**	**Continuous**				0.93	0.71	1.21	0.59
		2	3 (50.0%)	3 (50.0%)				
		3	7 (50.0%)	7 (50.0%)				
		4	16 (69.6%)	7 (30.4%)				
		5	15 (55.6%)	12 (44.4%)				
		6	8 (53.3%)	7 (46.7%)				
		7	6 (66.7%)	3 (33.3%)				
		8	2 (100.0%)	0 (0.0%)				
		9	1 (50.0%)	1 (50.0%)				
**Conflict of interest reported**	**Categorical**	Yes	32 (56.1%)	25 (43.9%)	0.74	0.32	1.68	0.47
		No	26 (63.4%)	15 (36.6%)	1	-	-	-
**Conflict of interest present**		Not reported	26 (63.4%)	15 (36.6%)	NA	NA	NA	NA
		No	32 (56.1%)	25 (43.9%)	NA	NA	NA	NA
**Funding reported**	**Categorical**	Yes	23 (56.1%)	18 (43.9%)	0.8	0.35	1.81	0.6
		No	35 (61.4%)	22 (38.6%)	1	-	-	-
**Type of orthodontic intervention**^**a**^	**Categorical**	1	41 (59.4%)	28 (40.6%)	1	-	-	-
		2	1 (100.0%)	0 (0.0%)	NA	NA	NA	NA
		3	16 (57.1%)	12 (42.9%)	1.1	0.45	2.67	0.84

^a^Type 1 orthodontic interventions: Orthodontic interventions to move teeth or change the jaw size or position for orthodontic purposes. Type 2 orthodontic interventions: Orthodontic interventions with additional surgical, pharmacological, or vibratory interventions. Type 3 orthodontic interventions: Orthodontic interventions to maintain or stabilize orthodontic results

Correct Table 6:

**Table 6:** Associations between presence of spin in the abstract and characteristics of the systematic review

**Item**	**Variable insertion in the model**	**Description**	**No (%)**	**Yes (%)**	**OR**	**Lower 95%CI**	**Upper 95%CI**	**P value**
**Journal**	**Categorical**	Cochrane	4 (40.0%)	6 (60.0%)	1	-	-	-
		EJO	18 (64.3%)	10 (35.7%)	0.37	0.08	1.63	0.19
		AJODO	14 (70.0%)	6 (30.0%)	0.29	0.06	1.39	0.12
		AO	14 (60.9%)	9 (39.1%)	0.43	0.09	1.95	0.27
		KJO	1 (33.3%)	2 (66.7%)	1.33	0.09	20.11	0.84
		O&C	7 (50.0%)	7 (50.0%)	0.67	0.13	3.45	0.63
**Year of publication**	**Continuous**				1.03	0.9	1.16	0.7
		2009	1 (100.0%)	0 (0.0%)				
		2010	1 (33.3%)	2 (66.7%)				
		2011	5 (71.4%)	2 (28.6%)				
		2012	2 (100.0%)	0 (0.0%)				
		2013	6 (66.7%)	3 (33.3%)				
		2014	3 (33.3%)	6 (66.7%)				
		2015	9 (75.0%)	3 (25.0%)				
		2016	5 (55.6%)	4 (44.4%)				
		2017	5 (55.6%)	4 (44.4%)				
		2018	6 (46.2%)	7 (53.8%)				
		2019	3 (75.0%)	1 (25.0%)				
		2020	7 (58.3%)	5 (41.7%)				
		2021	5 (62.5%)	3 (37.5%)				
**Number of authors**	**Continuous**				0.93	0.71	1.21	0.59
		2	3 (50.0%)	3 (50.0%)				
		3	7 (50.0%)	7 (50.0%)				
		4	16 (69.6%)	7 (30.4%)				
		5	15 (55.6%)	12 (44.4%)				
		6	8 (53.3%)	7 (46.7%)				
		7	6 (66.7%)	3 (33.3%)				
		8	2 (100.0%)	0 (0.0%)				
		9	1 (50.0%)	1 (50.0%)				
**Conflict of interest reported**	**Categorical**	Yes	32 (56.1%)	25 (43.9%)	0.74	0.32	1.68	0.47
		No	26 (63.4%)	15 (36.6%)	1	-	-	-
**Conflict of interest present**		Not reported	26 (63.4%)	15 (36.6%)	NA	NA	NA	NA
		No	32 (56.1%)	25 (43.9%)	NA	NA	NA	NA
**Funding reported**	**Categorical**	Yes	23 (56.1%)	18 (43.9%)	0.8	0.35	1.81	0.6
		No	35 (61.4%)	22 (38.6%)	1	-	-	-
**Type of orthodontic intervention**^**a**^	**Categorical**	1	41 (59.4%)	28 (40.6%)	1	-	-	-
		2	1 (100.0%)	0 (0.0%)	NA	NA	NA	NA
		3	16 (57.1%)	12 (42.9%)	1.1	0.45	2.67	0.84

^a^Type 1 orthodontic interventions: Orthodontic interventions to move teeth or change the jaw size or position for orthodontic purposes. Type 2 orthodontic interventions: Orthodontic interventions with additional surgical, pharmacological, or vibratory interventions. Type 3 orthodontic interventions: Orthodontic interventions to maintain or stabilize orthodontic results

The original article has been corrected.
