# Supplementary material for: Spin on adverse effects in abstracts of systematic reviews of orthodontic interventions: a cross-sectional study (part 2)
Source: Syst Rev. 2023 Jun 20;12:99. doi: 10.1186/s13643-023-02269-3 (PMC10280878; doi:10.1186/s13643-023-02269-3)
Supplement: Supplementary file 2 — Additional file 2. 2A. Differences between the protocol and the completed cross-sectional study. 2B. Search terms and their derivatives. 2C. Data collection forms. 2D. Adverse effects hypothetically linked to orthodontic interventions. [file 13643_2023_2269_MOESM2_ESM.docx]

**Additional file 2**

**Table of contents for additional file 2**

| **Page(s)** | **Additional file item** | **Description** |
| --- | --- | --- |
| 1 | Additional file 2A | Differences between the protocol and the completed cross-sectional study |
| 2 | Additional file 2B | Search terms and their derivatives |
| 3-6 | Additional file 2C | Data collection forms |
| 7-8 | Additional file 2D | Adverse effects hypothetically linked to orthodontic interventions |
| 9-10 | References | References for additional file 2 |

**Additional file 2A. Differences between the protocol and the completed cross-sectional study**

| **Differences between the protocol and the completed cross-sectional study** | **Rationale** |
| --- | --- |
| Fine-tuning of the definitions of the 3 types of spin | In our protocol we wanted to identify spin by comparing what was reported in the abstract with what was reported in the main text of the manuscript. We changed this to comparing what was reported in the abstract with what was found in the review. This fine-tuning was necessary, because on a few occasions what was reported in the main text of the review was not completely congruent with the findings of the review or pertinent information was not reported in the main text of the review, but only in supplementary files. |
| Explorative analyses to assess the presence of spin in the abstract and a series of predictors | We assessed the association between the presence of spin in the abstract and a series of predictors. These predictors were not defined a priori in our protocol, but several were explored in recent studies on spin in the field of orthodontics (Guo 2021, Makou 2021). We therefore conducted explorative analyses to determine associations between the presence of spin in the abstract and various predictors. |

**Additional file 2B. Search terms and their derivatives**

**Table. Search terms and their derivatives**

| **Search terms and their derivatives** | **Search terms for searching multiple words in a PDF** |
| --- | --- |
| “adverse” | ADVERSE, Adverse, adverse |
| “effect”, “effects” | EFFECT, Effect, effect |
| “reaction”, “reactions” | REACTION, Reaction, reaction |
| “complication”, “complications”, “complicated”, “complicating” | COMPLICAT, Complicat, complicat |
| “harm”, “harms”, “harmful” | HARM, Harm, harm |
| “risk”, “risks”, “risky” | RISK, Risk, risk |
| “safe”, “safety” | SAFE, Safe, safe |
| “side” | SIDE, Side, side |
| “toxic”, “toxicity” | TOXIC, Toxic, toxic |
| “benefit”, “benefits” | BENEFIT, Benefit, benefit |
| “result”, “results” | RESULT, Result, result |
| “finding”, “findings” | FINDING, Finding, finding |
| “outcome”, “outcomes” | OUTCOME, Outcome, outcome |
| “limitation”, “limitations”, limit | LIMIT, Limit, limit |
| “damage”, “damages”, “damaging” | DAMAGE, Damage, damage |
| “data” | DATA, Data, data |
| “information” | INFO, Info, info |
| “conflict”, “conflicts”, “conflicting” | CONFLICT, Conflict, conflict |
| “negative” | NEGATIVE, Negative, negative |
| “detrimental” | DETRIMENTAL, Detrimental, detrimental |
| “disadvantage”, “disadvantages”, “disadvantageous” | DISADVAN, Disadvan, disadvan |
| “down” | DOWN, Down, down |
| “injury”, “injuries”, “injured”, “injurious” | INJUR, Injur, injur |
| “byproduct”, “byproducts” | BYPRODUCT, Byproduct, byproduct |
| “collateral” | COLLATERAL, Collateral, collateral |
| “unfavorable”, “unfavourable” | UNFAVO, Unfavo, unfavo |
| “destructive” | DESTRUCT, Destruct, destruct |
| “unsafe” | UNSAFE, Unsafe, unsafe |
| “undesired”, “undesirable” | UNDESIR, Undesir, undesir |
| “recommend”, “recommendation”, “recommending” | RECOMMEND, Recommend, recommend |
| “emergency”, “emergencies” | EMERGEN, Emergen, emergen |

**Additional file 2C. Data collection forms**

**Data collection form on reporting of adverse effects in the abstract of systematic reviews of orthodontic interventions**

| **Items** | **Description** |
| --- | --- |
| Did the review seek any findings related to adverse effects of interventions in the included studies? | Answer: Yes/No  Yes: Any findings related to adverse effects of interventions in the included studies were sought by the reviewers.  Seeking any findings related to adverse effects of interventions in the included studies refers to reporting anywhere in the review (except in the Abstract) that such adverse effects in the included studies were sought.  Yes: Yes is also scored when reviewers only reported findings related to adverse effects of interventions in the included studies, but did not report that they actually sought them or planned to seek them. For example ‘Yes’ will be scored when outcomes on adverse effects of interventions in the included studies were reported in the review, but were not defined as objectives of the review.  Yes: Yes is also scored when the reviewers reported that they planned to seek (for example in the research objectives) findings related to adverse effects of interventions in the included studies, but did not report on these findings.  No: Findings related to adverse effects of interventions in the included studies were not sought by the reviewers. |
| In abstracts of systematic reviews of orthodontic interventions were potential adverse effects of these interventions reported or considered (i.e., discussed, weighed etc.)? | Answer: Yes/No  Yes: In abstracts of systematic reviews of orthodontic interventions potential adverse effects of these interventions were reported or considered (i.e., discussed, weighed etc.).  No: In abstracts of systematic reviews of orthodontic interventions potential adverse effects of these interventions were not reported or considered (i.e., discussed, weighed etc.). |

**Data collection forms to identify spin of adverse effects of orthodontic interventions in abstracts of systematic reviews**

To assign spin of adverse effects of orthodontic interventions in abstracts of systematic reviews we developed separate checklists for reviews that sought adverse effects of interventions and those that did not. These checklists were pilot-tested a priori during the development of our protocol. Because only 2 systematic reviews in this pilot study reported on adverse effects of interventions, we decided to conduct an additional pilot test to further fine-tune these checklists. Ten randomized controlled trials (RCTs) (2 per journal) of orthodontic interventions were selected consecutively from the websites of the 5 leading orthodontic journals from June 2021 backwards. RCTs were eligible for the pilot study if they assessed adverse effects of orthodontic interventions. The first 2 RCTs for each journal that were identified during this search process were selected. The fine-tuned checklists are reported in the tables under here and differ slightly from those reported in our protocol (Steegmans 2019). Changes were made to (1) reduce inter-operator differences in assigning various types of spin and (2) make the descriptions of spin more congruent with those given in the literature (Boutron 2018, Haneef 2017, Lazarus 2015) and with the definition of spin (in the abstract) on adverse effects of interventions presented in our protocol (Steegmans 2019), i.e., ‘Incomplete or inadequate reporting, interpretation, or extrapolation (or a combination of these variables) of findings on adverse effects of interventions in the abstract that could be misleading for the reader’(See additional file 2A).

**Data collection form to identify spin in abstracts of reviews that did seek adverse effects of interventions**

| **Items for misleading reporting (in the abstract) on adverse effects of interventions** | **Score** |
| --- | --- |
| 1) Not reporting in the abstract on the results of adverse effects found in the review. | Yes/no |
| 2) Selective reporting in the abstract on the results of adverse effects found in the review. | Yes/no |
| *Summary score on the presence of misleading reporting (in the abstract) on adverse effects of interventions* | *Yes/no*  *Yes is scored when one or more of the 2 items is answered with a ‘Yes’*  *No is scored when both items are answered with a ‘No’* |
|  |  |
| **Items for misleading interpretation (in the abstract) on adverse effects of interventions** |  |
| 1) Claiming in the abstract that the intervention is safe (has no or minimal adverse effects), despite concerning results on adverse effects found in the review e.g., based on non-statistically significant results on adverse effects with wide confidence intervals (Yavchitz 2016) | Yes/no |
| 2) Downgrading in the abstract the importance of the adverse effects, despite concerning results on adverse effects found in the review. | Yes/no |
| 3) Recommendations are made in the abstract for clinical practice that are not supported by the findings in the review on adverse effects’(Yavchitz 2016) | Yes/no |
| *Summary score on the presence of misleading interpretation (in the abstract) on adverse effects of interventions* | *Yes/no*  *Yes is scored when one or more of the 3 items is answered with a ‘Yes’*  *No is scored when all 3 items are answered with a ‘No’* |
|  |  |
| **Items for misleading extrapolation (in the abstract) on adverse effects of interventions** |  |
| 1) Results are extrapolated in the abstract to another population, intervention, outcome or setting than were assessed in the review despite evidence on adverse effects on a different population, intervention, outcome or setting. | Yes/no |
| *Summary score on the presence of misleading extrapolation (in the abstract) on adverse effects of interventions* | *Yes/no*  *Yes is scored when the item is answered with a ‘Yes’*  *No is scored when the item is answered with a ‘No’* |

**Data collection forms to identify spin in abstracts of reviews that did not seek adverse effects of interventions**

| **Items for misleading reporting (in the abstract) on adverse effects of interventions** | **Score** |
| --- | --- |
| 1) Reporting on results of adverse effects in the abstract when adverse effects were not sought. | Yes/no |
| 2) Reporting in the abstract that adverse effects were sought when they were not sought. | Yes/no |
| *Summary score on the presence of misleading reporting (in the abstract) on adverse effects of interventions* | *Yes/no*  *Yes is scored when one or more of the 2 items is answered with a ‘Yes’*  *No is scored when both items are answered with a ‘No’* |
|  |  |
| **Items for misleading interpretation (in the abstract) on adverse effects of interventions** |  |
| 1) Claiming in the abstract that the intervention is safe (has no or minimal adverse effects) despite not having sought adverse effects. | Yes/no |
| 2) Downgrading in the abstract the importance of the adverse effects, despite not having sought adverse effects. | Yes/no |
| 3) Recommendations are made in the abstract for clinical practice despite not having sought adverse effects. | Yes/no |
| *Summary score on the presence of misleading interpretation (in the abstract) on adverse effects of interventions* | *Yes/no*  *Yes is scored when one or more of the 3 items is answered with a ‘Yes’*  *No is scored when all 3 items are answered with a ‘No’* |
|  |  |
| **Items for misleading extrapolation (in the abstract) on adverse effects of interventions** |  |
| 1) Results are extrapolated in the abstract to another population, intervention, outcome or setting than were assessed in the review despite not having sought adverse effects. | Yes/no |
| *Summary score on the presence of misleading extrapolation (in the abstract) on adverse effects of interventions* | *Yes/no*  *Yes is scored when the item is answered with a ‘Yes’*  *No is scored when the item is answered with a ‘No’* |

**Additional file 2D. Adverse effects hypothetically linked to orthodontic interventions (Steegmans submitted 2022)***

| **Adverse effects related to** | **Description** |
| --- | --- |
| Tooth structures | Tooth crown   - decalcifications, decays, tooth wear, enamel cracks and fractures; discolorations, deterioration of prosthetic crown (as fracturing a ceramic one during debonding); - iatrogenic damage to the crown, e.g., fracture as a result of trauma   Tooth root   - root resorption, early closure of root apex, ankylosis; - iatrogenic damage to the root, e.g., fracture as a result of trauma   Tooth pulp   - ischemia, pulpitis, necrosis - iatrogenic damage to the pulp, e.g., fracture as a result of trauma |
| Periodontal tissues | - gingivitis, periodontitis, gingival recession or hypertrophy, alveolar bone loss, dehiscences, fenestrations, interdental fold, dark triangles; tooth mobility, plague retention, bacterial count |
| Intraoral (non-tooth or periodontal) tissues | - intraoral tissue irritations and inflammation such as mucosal ulcerations or hyperplasia or irritations of the tongue (as a result of trauma by appliances, e.g., breakage, failure, loosening etc. of appliances or long arch wires) - Scar formation after suturing - chemical burns (e.g., etching related) - thermal injuries (e.g., overheated burs) - nerve damage - tooth eruption, i.e., eruption disturbances (e.g., impactions) caused by orthodontic appliances |
| Extraoral tissues (non-temporomandibular tissues) | - cutting of lips or cheeks, eye injury (e.g., as a result of trauma by appliances, e.g., breakage, failure, loosening etc. of appliances or long arch wires or headgear-related trauma) - discomfort on the lip |
| Temporomandibular tissues and disorders | - temporomandibular tissues and disorders |
| Appliance failure | - breakage, failure, loosening etc. of appliances - long archwires, headgear-related trauma |
| Undesired treatment results | - inadequate morpho-functional, aesthetic or functional final result - inaccuracy of the treatment result - non predictability of the treatment result - Dental side effects e.g., unwanted tipping of teeth, anchorage loss etc. - Skeletal side effects, e.g., unwanted backward rotation of the mandible |
| Relapse and stability | - Relapse and stability of the obtained treatment result |
| Undesired qualitative experiences by the patient or carer(s) | Pain and discomfort   - orthodontic tooth movement-related pain and discomfort - appliance (intervention)-related pain and discomfort: i.e., pain and discomfort as a result of the appliance (intervention) itself with or without pain and discomfort associated with tooth movement e.g., tension or pressure of the appliances (constriction of appliances), speech difficulties, eating difficulties, swallowing difficulties, food accumulation, bad tastes and smells - additional intervention-related pain and discomfort, e.g., surgical and non-surgical adjunctive interventions to accelerate tooth movement   Tolerability/acceptance/stress issues with the treatment procedures   - Absence from work or studies and difficulties in daily activities - collaboration (compliance) issues or failure to complete treatment, e.g., dropout - patient anxiety - being teased - social discomfort - embarrassment to wear the appliance - behavioral changes of patients and parents, impaired family relationships - aesthetic look discontents during orthodontic appliance usage - concentration difficulties - reduced enjoyment of food and change in taste - sleeping difficulties - removal of appliance during sleep - development of mannerisms   Satisfaction with the treatment procedures and final result   - not satisfied with the treatment procedures (Check in text what was measured, i.e., during or after) - not satisfied with the final treatment result (Check in text what was measured, i.e., during or after) |
| Gastro-intestinal | - accidental swallowing of small parts of the orthodontic device (tubes, brackets); |
| Allergy | - Allergies to nickel or latex; |
| Cardio | - infective endocarditis; |
| Chronic fatigue |  |
| Cross infections | - from doctor to patient, patient to doctor, patient to patient. |
| Non-defined | Adverse effects that were not defined by the authors of the review: referring to ‘any adverse effect’, ‘any side effect’ etc. |
| Additional adverse effects | Additional adverse effects that were identified during data extraction that could not be labeled under any of the categories of adverse effects given in this table |

*Modified from Preoteasa et al. (Preoteasa 2012)

**References for additional file 2**

**Boutron 2018**

[Boutron I](https://www.ncbi.nlm.nih.gov/pubmed/?term=Boutron%20I%5BAuthor%5D&cauthor=true&cauthor_uid=29531025), [Ravaud P](https://www.ncbi.nlm.nih.gov/pubmed/?term=Ravaud%20P%5BAuthor%5D&cauthor=true&cauthor_uid=29531025). Misrepresentation and distortion of research in biomedical literature. [Proc Natl Acad Sci U S A.](https://www.ncbi.nlm.nih.gov/pubmed/29531025) 2018 Mar 13;115(11):2613-2619. doi: 10.1073/pnas.1710755115.

**Guo 2021**

Guo F, Fang X, Li C, Qin D, Hua F, He H. The presence and characteristics of 'spin' among randomized controlled trial abstracts in orthodontics. Eur J Orthod. 2021 Oct 4;43(5):576-582. doi: 10.1093/ejo/cjab044. PMID: 34397084.

**Haneef 2017**

Haneef R, Yavchitz A, Ravaud P, Baron G, Oranksy I, Schwitzer G, Boutron I. [Interpretation of health news items reported with or without spin: protocol for a prospective meta-analysis of 16 randomised controlled trials.](https://www.ncbi.nlm.nih.gov/pubmed/29151047) BMJ Open. 2017 Nov 17;7(11):e017425. doi: 10.1136/bmjopen-2017-017425.

**Lazarus 2015**

[Lazarus C](https://www.ncbi.nlm.nih.gov/pubmed/?term=Lazarus%20C%5BAuthor%5D&cauthor=true&cauthor_uid=26462565), [Haneef R](https://www.ncbi.nlm.nih.gov/pubmed/?term=Haneef%20R%5BAuthor%5D&cauthor=true&cauthor_uid=26462565), [Ravaud P](https://www.ncbi.nlm.nih.gov/pubmed/?term=Ravaud%20P%5BAuthor%5D&cauthor=true&cauthor_uid=26462565), [Boutron I](https://www.ncbi.nlm.nih.gov/pubmed/?term=Boutron%20I%5BAuthor%5D&cauthor=true&cauthor_uid=26462565). Classification and prevalence of spin in abstracts of non-randomized studies evaluating an intervention. [BMC Med Res Methodol.](https://www.ncbi.nlm.nih.gov/pubmed/?term=Classification+and+prevalence+of+spin+in+abstracts+of+non-randomized+studies+evaluating+an+intervention) 2015 Oct 13;15:85. doi: 10.1186/s12874-015-0079-x.

**Lefebvre 2021**

Lefebvre C, Glanville J, Briscoe S, Littlewood A, Marshall C, Metzendorf M-I, Noel-Storr A, Rader T, Shokraneh F, Thomas J, Wieland LS. Chapter 4: Searching for and selecting studies. In: Higgins JPT, Thomas J, Chandler J, Cumpston M, Li T, Page MJ, Welch VA (editors). *Cochrane Handbook for Systematic Reviews of Interventions*version 6.2 (updated February 2021). Cochrane, 2021. Available from [www.training.cochrane.org/handbook](http://www.training.cochrane.org/handbook).

**Makou 2021**

Makou O, Eliades T, Koletsi D. Reporting, interpretation, and extrapolation issues (SPIN) in abstracts of orthodontic meta-analyses published from 2000 to 2020. Eur J Orthod. 2021 Mar 19:567-575. doi: 10.1093/ejo/cjab009. Epub ahead of print. PMID: 33740054.

**Preoteasa 2012**

Preoteasa CT, Ionescu E, Preoteasa E. Chapter 18: Risks and complications associated with orthodontic treatment. In: Bourzgui F. (editor). Orthodontics-Basic aspects and clinical considerations. March 9, 2012 under CC BY 3.0 license. www.intechopen.com. [online] Available from:

<https://cdn.intechopen.com/pdfs/31388/InTech-Risks_and_complicationsassociated_with_orthodontic_treatment.pdf> (accessed December 4^th^ 2021).

**Steegmans 2019**

Steegmans, P.A.J., Di Girolamo, N, Meursinge Reynders, R.A. Spin in the reporting, interpretation, and extrapolation of adverse effects of orthodontic interventions: protocol for a cross-sectional study of systematic reviews. *Res Integr Peer Rev* **4,**27 (2019). <https://doi.org/10.1186/s41073-019-0084-4>

**Steegmans 2022 submitted to Systematic Reviews:** Seeking adverse effects in systematic reviews of orthodontic interventions: a cross-sectional study (Part 1).

**Yavchitz 2016**

[Yavchitz A](https://www.ncbi.nlm.nih.gov/pubmed/?term=Yavchitz%20A%5BAuthor%5D&cauthor=true&cauthor_uid=26845744), [Ravaud P](https://www.ncbi.nlm.nih.gov/pubmed/?term=Ravaud%20P%5BAuthor%5D&cauthor=true&cauthor_uid=26845744), [Altman DG](https://www.ncbi.nlm.nih.gov/pubmed/?term=Altman%20DG%5BAuthor%5D&cauthor=true&cauthor_uid=26845744), [Moher D](https://www.ncbi.nlm.nih.gov/pubmed/?term=Moher%20D%5BAuthor%5D&cauthor=true&cauthor_uid=26845744), [Hrobjartsson A](https://www.ncbi.nlm.nih.gov/pubmed/?term=Hrobjartsson%20A%5BAuthor%5D&cauthor=true&cauthor_uid=26845744), [Lasserson T](https://www.ncbi.nlm.nih.gov/pubmed/?term=Lasserson%20T%5BAuthor%5D&cauthor=true&cauthor_uid=26845744), [Boutron I](https://www.ncbi.nlm.nih.gov/pubmed/?term=Boutron%20I%5BAuthor%5D&cauthor=true&cauthor_uid=26845744). A new classification of spin in systematic reviews and meta-analyses was developed and ranked according to the severity. [J Clin Epidemiol.](https://www.ncbi.nlm.nih.gov/pubmed/?term=A+new+classification+of+spin+in+systematic+reviews+and+meta-analyses+was+developed+and+ranked+according+to+the+severity) 2016 Jul;75:56-65. doi: 10.1016/j.jclinepi.2016.01.020. Epub 2016 Feb 2.
